# Dreaming while awake: The beneficial effects of yoga Nidra on mental and physical recovery in two elite karate athletes

**DOI:** 10.1016/j.heliyon.2024.e24180

**Published:** 2024-01-05

**Authors:** Selenia di Fronso, Claudio Robazza, Dario Pompa, Maurizio Bertollo

**Affiliations:** a Behavioral Imaging and Neural Dynamics (BIND) Center, University “G. d’Annunzio” of Chieti-Pescara, Chieti, Italy; b Department of Medicine and Aging Sciences, University “G. d’Annunzio” of Chieti-Pescara, Chieti, Italy

**Keywords:** Arousal, iAPF, Interoceptive awareness, Recovery-stress balance, Relaxation, Sleep quality, Stress perception

## Abstract

Yoga Nidra (YN) naturally stimulates a hypnagogic state wherein an individual is physiologically asleep yet maintains a certain awareness to follow a guide's instructions. The aim of this study was to investigate the effects of this aware sleep state on recovery-stress balance in two elite karate athletes adopting an idiosyncratic and multimodal approach. One male and one female athlete underwent a YN intervention. Before intervention, after intervention and three weeks later, recovery-stress balance specific scales, perceived stress, cognitive and somatic anxiety, subjective and objective sleep quality, and individual alpha peak frequency (iAPF) values were assessed. Perceived quality of recovery was continuously monitored for three months including the period of the investigation. Feelings and arousal levels before and after each YN session were also examined. Our results showed a YN general positive effect; however, the intervention had higher sport specific effects in the male compared to the female athlete. On the other hand, in the female athlete, YN seems to have effects both from an emotional and physical point of view. We also noted the intertwined relationship among interoception, perceived stress and YN effects. Also, findings suggest that iAPF modulation reflected improved recovery skills or a better control of stressful situations, while the acute effects on arousal levels were expression of anxiety or energy reduction. Overall, YN improved both the perceived quality of recovery and sleep quality, shedding light on the importance of YN for recovery-stress balance enhancement in the sport context.

## 1 Introduction

Recovery-stress balance is crucial to reach and maintain a state of well-being and counteract physical and mental stressors which may threaten sport performance and, most importantly, athletes' health [1]. To this end, recovery is overall described as a pro-active, multidimensional phenomenon crucial to replenish physical and mental resources [2] and prevent biopsychosocial imbalances in athletes [3]. Athletes, especially the elite ones, often face a great deal of mental, emotional and social demands; moreover, they often sleep less than the recommended night-time sleep duration (i.e.,7–9 h per night) [4]. Factors like evening high-intensity training, long-haul travels to competition venues, pre-competition anxiety or mental fatigue can negatively influence not only duration but also sleep quality in athletes [5]. As a consequence, poor sleep can cause attention depletion or concentration disruption, thereby leading to self-regulation and recovery deficits [6]. In turn, these deficits negatively impact athletes’ emotion regulation and stress levels. Of note, the relationship between sleep and stress in not univocal, as studies have shown that stress negatively affects sleep [4]. Several stressors (e.g., psychological, social, physiological) may actually hamper adequate sleep. For example, it was shown that higher levels of mental strain were associated with reduced sleep duration and efficiency (i.e., the percentage a person sleeps, in relation to the amount of time a person spends in bed) in junior endurance athletes [7]. These results extend to the young athletic population the notion that psychological stress can have harmful effects on sleep [8]. Accordingly, the bi-directional relationship between sleep and stress deserves greater attention, especially in elite sport settings [4].

Empirical evidence suggests sufficient sleep is one of the most obvious strategies for enhancing recovery [9,10], managing physical/mental fatigue and defending athlete well-being [1]. Consequently, further investigating methods that boost sleep quantity and, more importantly, sleep quality, can be of great importance in the sport context. Among the strategies and methods for boosting sleep and recovery we can include relaxation techniques (e.g., mindfulness based meditations), as they improve recovery skills, reduce muscle tension and positively impact autonomic nervous system (e.g., boosting parasympathetic activation, slowing and/or regulating breathing rhythm and decreasing heart rate) [11]. In this regard, a brief mindfulness induction on university athletes’ sleep following evening training was found to reduce pre-sleep arousal levels and increase overall rest and sleep quality [12]. Also, Jones et al. [13] studied the effects of a mindfulness-based stress reduction (MBSR) programme on psychological well-being and subjective and objective sleep parameters in a sample of NCAA division female rowers. The authors noted that mindfulness improved both psychological well-being and sleep quality.

**Fig. 1:**
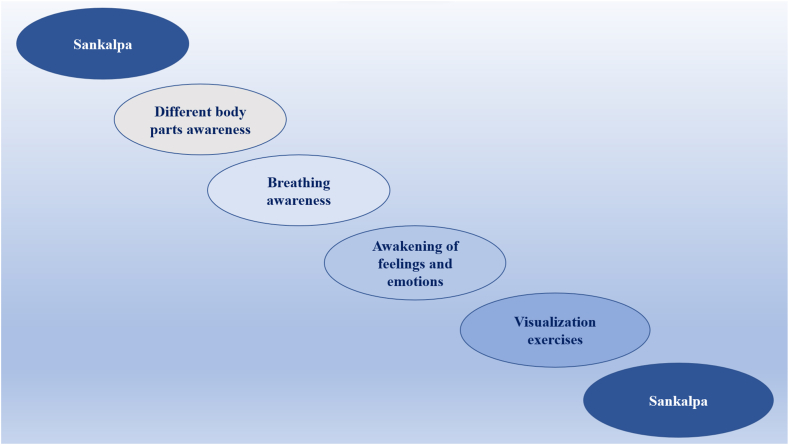
The main stages of a yoga Nidra session. Sankalpa: intention expressed as a simple, short, and positive sentence (e.g., “I am calm,” “I am relaxed”).

Drawing on the aforementioned importance of continued exploration of recovery strategies in sport context, a recent opinion paper claims the importance of yoga Nidra (YN) for athletes [14]; moreover, a systematic review of yoga interventions effects on psychological health of competitive athletes suggests that mind-body techniques, including YN, should be better studied [15]. YN, literally yoga sleep, is generally described as a simple meditation practice that can be used by everyone [16]. Overall, as illustrated in Fig. 1, YN includes a sequence of breathing, guided body awareness, and visualization exercises that make it a complete and systematic method to induce physical and mental relaxation [17,18]. Executed in supine position, YN naturally stimulates a hypnagogic state wherein an individual is physiologically asleep yet maintains a certain level of internal/external awareness [14]. Indeed, while participants practice detachment from most of the stimuli, the auditory channel remains receptive (bell sounds are indeed used throughout the practice) so that they can follow guide's instructions. This is why YN is also considered as an aware sleep state, a feature that distinguishes this from other meditation-based practices, such as transcendental meditation or body scan, which are mainly considered as aware awake states [19]. Another key component of YN sessions is called Sankalpa (see Fig. 1), the Sanskrit word for “intention.” It represents a personal resolution expressed as a simple, short, and positive sentence (e.g., “I am quiet,” “I am successful”). Its regular mental repetition stimulates cognitive restructuring processes “driving” the unconscious toward the desired state [16]. Sankalpa and cognitive restructuring processes can prevent or counteract dysfunctional cognitions, thereby inducing life satisfaction and individual well-being [20]. Moreover, due to a general parasympathetic dominance [21] and a subsequent high cardiac vagal control [22], YN interventions can significantly improve subjective and objective sleep quality [16,23]. Indeed, it is frequently suggested in sleep lab protocols [19], also because 1 h of YN can be as restorative and rejuvenating as 4 h of ordinary sleep [17].

Overall, there has been a recent and growing interest in YN effects, especially on perceived stress, well-being, insomnia and chronic sleep disorders. Regarding insomnia, for example, as demonstrated by Datta and colleagues [23], YN can improve different sleep parameters (e.g., wakening after sleep onset-WASO, sleep onset latency, total sleep time-TST) in the elderly. However, research regarding YN and athletes is still scarce, with mainly students, workers and veterans being considered [[24], [25], [26]]. Only in the archery context, this technique was found to help improve athletic performance both directly and indirectly by enhancing vigilance and by increasing training participation due to better physical and mental recovery, respectively [27].

By relying on the premises above, and grounded on the principles of translational medicine to improve health outcomes, the aim of this study was to investigate the effects of an aware sleep state on sleep quality and the usefulness of YN for improving recovery-stress balance in two elite karate athletes. We adopted an idiosyncratic approach to advancing our understanding of the mechanisms underlying recovery [28]. Each athlete evaluates their recovery differently and has different levels of stress [29]. Moreover, case studies allow researchers and practitioners to better identify principles and practice for both team and individual sports, and to develop procedures to assess intervention success [30]. Within a multimodal approach, we also measured a psychophysiological parameter, namely the individual alpha peak frequency (iAPF), to better establish the impact of YN on the alpha peak modulation [31]. This allows examination of the neural correlates of a passive recovery strategy. Moreover, iAPF was adopted as a neural marker to examine burnout in athletes from a psychophysiological perspective [32], thus corroborating its usefulness in recovery-stress balance settings.

In recent years, several studies have investigated the benefits of mindfulness in karate, a highly dynamic sport requiring effort and self-control often accompanied by high stress levels [33]. For example, Miyata et al. [34] investigated whether martial arts practice, combined with physical and mental practice, was related to mindfulness and psychological health. Findings showed that the use of this technique induced a better athletes' psychological state, and that the longer and more frequent the practice, the better the state. However, no research has been conducted on the impact of YN practice on sleep and recovery-stress balance in karate athletes. We hypothesized a general positive effect of YN on sleep quality and recovery-stress balance in both athletes. In particular, based on previous research on the relationship between stress and interoceptive awareness (IA) features [35], we discuss our results by also considering individuals’ interoception. Whether the athlete was a worker or a student was also taken into account. Additionally, we expected that the female athlete in our study would benefit more from YN intervention as a result of the general greater interest in alternative “therapies” expressed by women [36]. However, given the lack of studies on the usefulness of YN in the sport context, this research should be considered exploratory in nature and pave the way for new research lines.

## 2 Results

### 2.1 Subject 1 results

**Fig. 2:**
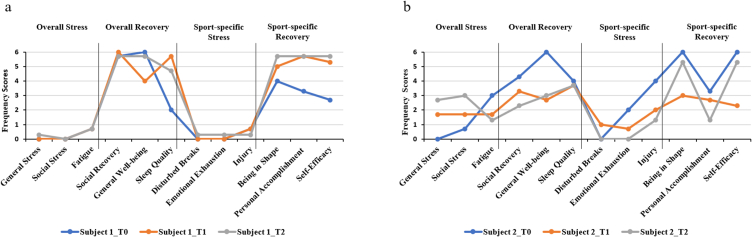
Scale scores of the RESTQ-Sport-36 in subject 1 (panel a) and subject 2 (panel b) before intervention (T0), after intervention (T1) and at follow up (T2).

Fig. 2 shows the profile of recovery-stress balance obtained by the male athlete of this study (subject 1) and thus the scores of the 36-item version of the Recovery-Stress Questionnaire for Italian athletes (RESTQ-Sport-36) [37] before the YN intervention (T0), after the intervention (T1) and three weeks later, i.e., at follow up (T2). Specifically, scale scores of *General Stress*, *Social Stress, Social Recovery, Fatigue, Disturbed breaks*, *Emotional Exhaustion*, and *Injury* remained fairly stable over time. On the other hand, while *General Well-being* scores decreased, especially at T1, *Sleep Quality*, *Being in Shape*, *Personal Accomplishment and Self-Efficacy* scores increased. With regards to the Italian version of the perceived stress scale (I-PPS) [38], despite subject 1 showed increased scores at T1, he can be considered as having low scores across all times of measurement (I-PPS scores at T0 = 1; T1 = 3; T2 = 1). The athlete's daily assessment through Total Quality Recovery scale (TQR) [39] showed scores ranging between reasonable and good recovery before intervention, and between good and very very good recovery during the intervention period. On the other hand, at the end of the intervention, recovery mainly ranged between poor and reasonable scores (see also Table 1S in supplementary materials).

**Table 1:** Actigraphy measures before intervention (T0), after intervention (T1), and at follow up (T2) in subject 1 and subject 2.

		Latency (min)	SE	TST (min)	WASO	N awaken	Avg awaken (min)
Subject 1	T0	6.2	95.74 %	450.6	13.2	9.2	1.43
	T1	5.4	96.26 %	374.8	9.4	5.6	1.68
	T2	7.5	92.39 %	355.25	22.75	15	1.52
Subject 2	T0	6.5	95.07 %	370.25	12.25	6.75	1.81
	T1	4.5	97.04 %	482.5	8.5	6.25	1.36
	T2	4.5	94.9 %	389.5	14.25	8.5	1.68

**Note.** Min = minutes; SE = Sleep efficiency; TST = Total sleep time; WASO = Wake after sleep onset; N awaken = Number of awakenings; Avg awaken = Mean length of awakenings. Subject 1 = Male elite karate athlete, 36yo; Subject 2 = Female elite karate athlete, 19yo.

*Cognitive arousal* score on the Pre-sleep arousal scale (PSAS-C) [40] was not high at T0 and slightly decreased at T1. The PSAS-C scores were 9 (T0), 8 (T1) and 9 (T2). *Somatic arousal* (PSAS-S) scores were also low (8) and slightly increased at T1 (9) and at T2 (10). This athlete also showed a total score of the Pittsburgh Sleep Quality Index (PSQI) [41] of 3, 4, 4 at T0, T1 and at T2, respectively. Despite the increase of this parameter, the athlete was able to maintain sleep quality under the cut-off score of 5 which usually discriminates good and poor sleepers [42]. As shown in Table 1, actigraphy measures, especially latency (i.e., the time required for the transition from wakefulness to the onset of sleep), sleep efficiency (SE), WASO, and number of awakenings improved at T1. On the contrary, the same parameters worsened at T2.

Table 2 shows the values of feeling scale (FS) [43] and felt arousal scale (FAS) [44] administered immediately after and before each YN session. Regarding the FS, subject 1 always felt well before the sessions, and YN often improved his feelings. As for FAS, its seems that YN was mostly able to lower arousal levels, expressed in terms of energy, in subject 1.

**Table 2:** Feeling scale (FS) and felt arousal scale (FAS) values experienced by subject 1 and subject 2 before and after each yoga Nidra session.

Subject 1										
	*Session1*	*Session2*	*Session3*	*Session4*	*Session5*	*Session6*	*Session7*	*Session8*	*Session9*	*Session10*
**FS_PRE**	3	4	4	4	3	4	3	4	4	3
**FS_POST**	3	5	4	4	3	4	4	5	5	4
**FAS_PRE**	3(energy)	2(energy)	4(energy)	2(energy)	4(energy)	3(energy)	2(energy)	3(energy)	2(energy)	3(energy)
**FAS_POST**	2(energy)	1	2(energy)	1	3(energy)	2(energy)	1	3(energy)	2(energy)	2(energy)
**Subject 2**										
	*Session1*	*Session2*	*Session3*	*Session4*	*Session5*	*Session6*	*Session7*	*Session8*	*Session9*	*Session10*
**FS_PRE**	3	−1	−2	−5	−3	−4	−3	0	0	3
**FS_POST**	3	0	0	−3	−1	−3	−2	1	0	3
**FAS_PRE**	6(energy)	2(anxiety)	3(anxiety)	2(anxiety)	1	2(anxiety)	1	2(anxiety)	3(energy)	2(energy)
**FAS_POST**	3(energy)	1	2(calmness)	1	1	1	1	1	2(energy)	1

**Note.** Feeling scale ranges from −5 (very bad) to +5 (very good); felt arousal scale ranges from 1 (low arousal) to 6 (high arousal); high/low levels of arousal can have both a positive (energy/calmness) or negative (anxiety/boredom) connotation. Subject 1 = Male elite karate athlete, 36yo; Subject 2 = Female elite karate athlete, 19yo.

Means and standard deviations of iAPF values of EEG data collected at T0, T1 and T2 during a mental arithmetic stress test are listed in Table 3. One-way analysis of variance (ANOVA) on iAPF values at T0 showed no differences [F(1692; 3221,540) = 0.015, *p* = 0.973, η_p_^2^ = 0.000, Power = 0.052] among specific phases of EEG data collection (i.e., baseline, stress and recovery). One-way ANOVA conducted on iAPF values at T1 showed significant differences [F(1998; 3804,097) = 22.391, *p* < 0.001, η_p_^2^ = 0.012, Power = 1] among phases. Post hoc pair-wise comparisons with Bonferroni correction showed significant differences between baseline and stress phase (*p* < 0.001, *d* = 0.11), between baseline and recovery phase (*p* = 0.021, *d* = 0.08), and between stress and recovery phase (*p* < 0.001, *d* = 0.21). Decreased iAPF average scores was observed over the course of stress phase, while an increase toward higher frequencies was shown during recovery. One-way ANOVA on iAPF values at T2 yielded no significant differences [F(1993; 3795,462) = 0.635, *p* = 0.529, η_p_^2^ < 0.001, Power = 0.157] among phases of the stress test.

**Table 3:** Means and (standard deviations) of individual alpha peak frequency (iAPF) values of EEG data collected before intervention (T0), after intervention (T1), and at follow up (T2) during baseline, stress and recovery phases of the arithmetic stress test performed by subject 1 and subject 2.

	T0			T1			T2		
**iAPF**	*Baseline*	*Stress*	*Recovery*	*Baseline*	*Stress*	*Recovery*	*Baseline*	*Stress*	*Recovery*
Subject 1	11.21 (1.24)	11.21 (1.24)	11.21 (1.23)	9.81 1.55	9.61 (1.49)	9.94 (1.52)	10.05 (1.51)	10.10 (1.56)	10.09 (1.54)
Subject 2	10.00 (1.32)	9.64 (1.42)	9.84 (1.24)	9.70 (1.17)	9.75 (1.27)	9.76 (1.25)	10.05 (1.28)	9.70 (1.38)	9.92 (1.26)

**Note.** Means and standard deviations are computed on ∼ 1920 each phase. Subject 1 = Male elite karate athlete, 36yo; Subject 2 = Female elite karate athlete, 19yo.

### 2.2 Subject 2 results

Fig. 2 shows the profile of recovery-stress balance obtained by the female athlete of this study (subject 2) and thus the scores of the 36-item version of the RESTQ-Sport-36 at T0, T1 and T2. In detail, the scores of *General Stress* and *Social Stress* increased across measurement times, while the scores of *Sleep Quality* and *Disturbed Breaks* remained fairly stable. On the other hand, the scores of *Social Recovery*, *General Well-being*, *Being in Shape*, *Personal Accomplishment*, *Self-Efficacy*, and, interestingly, *Fatigue*, *Emotional Exhaustion* and *Injury*, decreased. I-PPS scores decreased at T1 and increased again at T2 (I-PPS scores at T0 = 25; T1 = 20; T2 = 32). The athlete's daily assessment through the TQR scale showed scores ranging between reasonable and good recovery before intervention, and between good and very very good recovery during the intervention period. Conversely, at the end of the intervention, recovery showed a great range of variation (see also Table 1S).

PSAS-C was high at T0 but decreased at T1. Scores increased again at T2. Specific PSAS-C scores were 29 (T0), 23 (T1) and 26 (T2). On the other hand, PSAS-S scores were 14 (T0), 13 (T1) and 14 (T2). Subject 2 also showed a total PSQI score of 7, 5, 5 at T0, T1 and T2 respectively, showing an overall improvement in sleep quality. As reported in Table 1, similar to subject 1, actigraphy measures, including TST, improved at T1, while they worsened at T2.

As shown in Table 2, YN improved FS scores of subject 2. Regarding FAS, it seems that YN lowered arousal levels, expressed in terms of anxiety, of this athlete.

Means and standard deviations of iAPF values of EEG data collected at T0, T1 and T2 are listed in Table 3. One-way ANOVA on iAPF values at T0 yielded significant differences [F(1987; 3782,424) = 34.904, *p* < 0.001, η_p_^2^ = 0.018, Power = 1] among the different phases of EEG data collection (i.e., baseline, stress and recovery in the mental arithmetic test). Specifically, post hoc pair-wise comparisons with Bonferroni correction showed significant differences between baseline and stress phase (*p* < 0.001, *d* = 0.26), between baseline and recovery phase (*p* < 0.001, *d* = 0.12), and between stress and recovery phase (*p* < 0.001, *d* = 0.14). In particular, decreased iAPF average was observed over the course of stress phase, while an increase toward higher frequencies was evident during recovery. One-way ANOVA conducted on iAPF values at T1 showed no significant differences [F(1998; 3803,499) = 1.593, *p* = 0.204, η_p_^2^ = 0.001, Power = 0.339] among phases. One-way ANOVA on iAPF values collected at T2 showed significant differences [F(1985; 3778,818) = 34.201, *p* < 0.001, η_p_^2^ = 0.018, Power = 1] among phases of EEG data collection. Post hoc pair-wise comparisons with Bonferroni correction showed significant differences between baseline and stress phase (*p* < 0.001, *d* = 0.26), between baseline and recovery phase (*p* = 0.005, *d* = 0.10), and between stress and recovery phase (*p* < 0.001, *d* = 0.16). Similarly to T0, iAPF average decreased over the course of stress phase, while there was an increase toward higher frequencies during recovery.

## 3 Discussion

The aim of our study was to understand the role of YN in terms of recovery-stress balance in two elite karate athletes. Specifically, we investigated the effects of an aware sleep state on recovery-stress balance, perceived stress, cognitive and somatic anxiety, sleep quality, and iAPF values before intervention, after intervention and three weeks later. Perceived quality of recovery was also examined by monitoring it continuously for three months, including the period of our investigation. Feelings and arousal levels before and after each YN session were also considered.

### 3.1 Subject 1

Regarding recovery-stress balance scales, the results obtained showed that, despite competitions (and the increase in ratings of perceived exertion -RPE [45]- collected by the athletes’ coach; see also Table 1S), YN sessions benefited the male athlete, as an increase in stress and fatigue in general was not observed. These findings concur with the notion that YN practice serves as a physical and mental recovery strategy to counteract the effects of tight competition bouts over weeks and restore the system and mental balance [2,14]. Also, although it did not appear beneficial for well-being from an overall perspective, given the improvement of *Being in Shape, Personal Accomplishment* and *Self-Efficacy* scores, we believe that YN was likely effective in ameliorating sport-specific aspects of recovery. Findings are also in line with those found in other populations (e.g., cancer patients), according to which relaxation improve self-efficacy [46]. Furthermore, the intentions put into simple, short and positive sentences, repeated at the beginning and end of the sessions (i.e., Sankalpa), could have boosted personal accomplishment and self-efficacy processes toward the achievement of the desired state and goals [16].

The slight increase in perceived stress after intervention could be due to the fact that the scale utilized to measure this perception, although meaningful to athletes [34,47], is not sport specific but rather related to general aspects of individuals' life [48]. Thus, the athlete might have responded to the items by considering other aspects of his life beyond sport; in this regard, it should be mentioned that subject 1 was also a night shift worker. This type of work, along with the competition period, may have influenced his perception of stress [49]. However, as mentioned above, it can be assumed that he perceived low stress levels even after intervention. Thus, YN was probably a protective factor towards stress levels which otherwise would have been higher. Of note, this subject was characterized by high level of IA, which is a metacognitive measure of interoceptive ability [50], especially in terms of self-regulation and trusting (i.e., the experience of one's body as safe and trustworthy). Thus, grounded on previous literature, paying attention to body sensations (self-regulation) could have served to regulate the athlete’ perceived levels of stress [31]. Indeed, good self-regulation skills are generally associated with lower somatic and cognitive anxiety, stronger mental health [51], and higher resilience and coping abilities, leading to better control of stressful situations. Also, trusting skills may have represented a contributing factor in the regulation of perceived stress levels [35]. Importantly, guided body awareness exercises typical of YN may have amplified both self-regulation and trusting skills.

Athlete's daily assessment using the TQR scale lead us to argue that, despite the perception of recovery could be considered good even before starting the intervention, YN benefited the quality of recovery in subject 1. This is further highlighted by the fact that perception of recovery assessed daily also after intervention decreased and was sometimes poor. The positive impact of YN was apparent from the second session onward, as during the first session participants generally need to become familiar with the technique [23]. In particular, sessions 6 through 9 were characterized by maximal quality of recovery. This probably happened because, after five lessons, YN generally decreases anxiety and dysfunctional emotions [23]. Cognitive restructuring processes induced by YN practice may indeed regulate post-performance negative emotions that hinder recovery processes, thus stimulating emotional detachment [52]. These findings seem to be at odds with those of Coimbra and colleagues [53] who found mindfulness-based trainings did not affect recovery in volleyball players. This discrepancy could be due to the comprehensiveness of YN technique, which, through guided body awareness, visualization, and breathing exercises, can improve both physical and mental relaxation [17,18], having a greater impact on the quality of recovery.

The intervention had no effects on pre-sleep somatic arousal, but had a slight effect on cognitive arousal. Of note, subject 1 scored low on both parameters across all measurement times. Beyond parasympathetic dominance, a decrease in cognitive arousal, even if minimal, could explain the improvement in sleep quality [54]. It is likely that after YN intervention, this athlete, was less preoccupied before sleep. Indeed, cognitive arousal experienced before bedtime is one of the factors that interfere with the individual's ability to initiate and maintain sleep [54]. YN seemed to improve both self-assessed (e.g., sleep quality scale of RESTQ-Sport-36) and actigraphy-based sleep quality (e.g., latency, WASO). These findings are in line with those of Datta et al. [27] who found significant improvement in total wake duration and SE in the subjective sleep diary of an elite archer after a YN intervention. They also reinforce the findings of other authors who previously observed a significant improvement in SE and a decrease in WASO in MBSR participants [13]. On the contrary, we noted a reduction in TST. This probably happened because of this subject's particular work. However, it seems that his hours of sleep improved in terms of quality. As indicated by the values collected before and after each session, improvement in sleep quality in this athlete may also be ascribed to the fact that the intervention was able to maintain or reinforce his positive feelings and decrease arousal (energy) levels experienced before bedtime rather than simply induce calmness. These results partially reflect what we observed considering pre-sleep arousal levels from a chronic perspective, and the importance of practising yoga for psychological benefits [55].

Regarding iAPF values, the results after intervention may reflect a better modulation of alpha frequencies, improved recovery skills and a more effective recovery-stress balance [31]. The modulation of the iAPF following mental arithmetic stress test may be related to stress-induced activation of the brain's arousal mechanisms and is probably linked to enhanced alertness during performance [56]. We could argue that this increase in iAPF is necessary to enhance alertness in order to respond faster to threatening situations [32], similar to the shift towards higher frequencies that occurs after physical stress [31,57].

### 3.2 Subject 2

Regarding recovery-stress balance, the results showed that the YN intervention was not impactful on social stress. In this regard, the transition from high school to the first year of university of this athlete, along with the period of competitions, may have amplified this kind of stress [58]. As a consequence, YN did not benefit athlete's well-being from an overall perspective. However, in subject 2, the intervention likely influenced well-being from an emotional standpoint, in line with the idea that, as a relaxation practise, YN generally triggers women as they are more prone to be emotionally exhausted [59]. Despite feelings of being in shape generally decreased, YN, as a mental but also physical relaxation practise [14], likely relieved self-assessed fatigue, muscle pain/soreness and body aching problems.

Of note, this athlete was characterized by low levels of IA, especially in terms of trusting. This is probably one of the reasons why she perceived high levels of stress before intervention, after intervention and at follow up [35], especially when compared to subject 1. However, perceived stress decreased after YN intervention. This may be due to the training/development of IA skills following YN, encompassing identifying, accessing, and appraising internal bodily signals, which are crucial components for emotion and stress regulation [60].

The athlete's daily assessment using the TQR scale lead us to argue that YN had a positive influence on perceived quality of recovery. Similar to subject 1, this positive influence was particularly evident from the second session onward, reinforcing the need to become familiar with the technique during the first session [23]. In particular, the perceived quality of recovery was maximal especially the morning after YN sessions, which could underscore the beneficial influence of this technique on sleep. Observing the quality of recovery after intervention, we noted that it remained very good during the first three days, but then settled down to poor levels (e.g., 4th of May). These data may recall the importance of mind-body practise in facilitating recovery processes [1,14].

After YN intervention there was a slight decrease in the somatic arousal experienced before bedtime. Hence, the athlete likely experienced less anxiety related to increased heart rate, cold feelings in her body, stomach ache, etc. Importantly, we noted also a reduction in pre-sleep cognitive arousal after YN intervention. It is worth to mention that previous studies in non-clinical samples have identified cut-off scores for the PSAS-C and PSAS-S of 20 and 14, respectively [61]. Thus, the chance of developing insomnia-related issues also decreased. Improvements in both somatic and cognitive arousal before falling asleep may have synergistically influenced sleep quality too. Of note, the RESTQ scores suggest a reduction in sleep quality. However, given the problems this specific scale might have, as reported by di Fronso et al. [37], we relied on sleep quality based on Pittsburgh and actigraphy data. Regarding self-assessed sleep quality Pittsburgh-derived, we can argue that this athlete, after intervention, can be considered as a good sleeper. This is confirmed also by actigraphy measures, with improvements, for example, in sleep latency which supports the usefulness of this strategy on the ability to sleep on demand (see di Fronso et al.) [14]. Once initiated, sleep was also less interrupted and more efficient. The discrepancy between self-assessed and actigraphy-based sleep quality at follow up would deserve greater attention. As indicated by the values collected before and after each session, improvement in sleep (and recovery) quality in this athlete may also be related to the slight but frequent improvement in FS values and, in particular, to the decrease of arousal levels expressed by the athlete in terms of anxiety reduction.

Regarding iAPF values, the results after intervention, while probably not indicative of improved recovery skills and a more effective recovery-stress balance as in subject 1, may reflect an improved control of stressful situations [62]. In subject 2, indeed, we noted a better modulation of the iAPF during the arithmetic stress phase, reinforced by the fact that before YN intervention and at follow up, over the course of the stress test, there was a shift of this parameter towards lower frequencies.

## 4 Conclusion

### 4.1 Limitations

A limitation of our study is that it was conducted on only two elite participants. Another limitation is that YN was not compared with other recovery strategies. Moreover, only perceived quality of recovery and ratings of perceived exertion were monitored continuously, whereas the other measures were mostly collected before intervention, after intervention and at follow up. Future studies should involve large samples, randomized controlled trials and envisage protocols that allow for gender, competitive level, type of sport and mind-body technique comparisons. Furthermore, a more comprehensive EEG data collection before and after a YN programme could allow to properly capture neural efficiency, neural proficiency and hypo frontality hypotheses that better depicts athletes’ mental states. Moreover, heart rate variability (HRV) data and ad hoc trials could be useful to clarify the nature of changes in HRV, as well as the physiological mechanisms underlying the increase in HRV induced by YN.

### 4.2 Strengths

On the other hand, according to Ericsson [28], an in-depth individual examination of elite athletes provides a lot of useful information. Additionally, we used validated scales, and although we were able to collect alpha oscillations only, we adopted a psychophysiological perspective that, to date, has rarely been used to inform athletes and coaches about stress and recovery processes. We also considered the acute effects of the intervention, which made the investigation more comprehensive. Also, studying YN in the sport context can be considered a novelty as the effects of an aware sleep state on sleep quality in athletes were scantly investigated. In this study we did not consider performance, yet both athletes qualified for the 2022 summer world championship, with one of them winning the gold medal in their category. Thus, future investigations should consider the relationship among recovery-stress balance, performance and YN effects. In addition, future research should examine the reasons why results do not persist over time, such as the duration of the intervention and the administration modality.

### 4.3 Concluding remarks

Our results mostly confirmed the general hypothesis of the study but also turned the spotlights on some YN effects that deserve further attention. In particular, we highlight that the intervention had higher sport-specific effects in the male athlete, and that in the female athlete YN seems to have both emotional and physical effects. Physical improvements in the pre-sleep somatic arousal levels were also noted in the female athlete. The intertwined relationship among IA, perceived stress and YN effects was also highlighted. In particular, it seems that the lower the trusting, the higher the perceived stress; However, the lower trusting, the higher YN effects on perceived stress, probably because of a larger margin of improvement in IA skills. Additionally, iAPF modulation can reflect improved recovery skills or better control of stressful situations, and the acute effects (after each yoga session) on arousal levels can be expressed in terms of reduced anxiety or energy. Of note, the intervention seems effective in ameliorating the quality of both perceived recovery and sleep, further shedding light on the importance of using YN in the sport context to enhance recovery-stress balance. Thus, practitioners should consider deepening their holistic approach to sports coaching and learn (new) mind–body techniques useful for athletes’ health.

## 5 Methods

### 5.1 Participants

One male (subject 1) and one female (subject 2) karate athlete, aged 36 and 19 years respectively, volunteered in our study. They had no neurological and/or psychological diseases and did not use drugs. They had no hypersomnia problems and daytime sleepiness issues. They were members of the FESIK (Federazione Educativa Sportiva Italiana Karate) national team and had participated in several major international events, including European and World championships. In particular, at the time of the present investigation, they were qualifying for the upcoming Karate WUKF (World Union of Karate Do Federation) Championship of Fort Lauderdale (Florida). They had no previous experience with YN. Subject 1 was a night shift worker and had the following levels of IA components: Attention Regulation = 4.6; Emotional Awareness = 4; Self-Regulation = 5; Trusting = 5. Subject 2 was attending the first year of university and had the following levels of IA components: Attention Regulation = 2.1; Emotional Awareness = 4; Self-Regulation = 3.3; Trusting = 0.7. After learning the purposes of the study, they agreed to participate and signed a written informed consent. The study was approved by the Ethics Committee for Biomedical Research of Chieti-Pescara University (ref. n. 17-15/07/2021), and was undertaken in compliance with the Declaration of Helsinki and the international principles governing research on humans.

In our study we used the following questionnaires:

*RESTQ-Sport-36*. The 36-item Recovery-Stress Questionnaire (RESTQ-Sport-36) for Italian athletes [37] was used to assess recovery-stress balance. It includes 12 scales consisting of 3 items each plus one “warm up” item not included in the scoring. The 12 scales are conceptually included into 4 categories—Overall Stress, Overall Recovery, Sport-specific Stress, and Sport-specific Recovery—consisting of 3 scales each [37]. Specifically, General Stress, Social Stress, and Fatigue pertain to the Overall Stress category, while Social Recovery, General Well-being, and Sleep Quality pertain to the Overall Recovery. Disturbed Breaks, Emotional Exhaustion, and Injury are included in the Sport-specific Stress category, while Being in Shape, Personal Accomplishment, and Self-Efficacy are included in the Sport-specific Recovery category. Participants are asked to indicate how often they were involved in specific activities during the past three days/nights using a 7-point Likert-type scale ranging from 0 (never) to 6 (always). According to RESTQ-Sport-36 manual, without essential changes in the internal consistency of the scales, the time frame can be extended up to two weeks. We used this time frame to better align this questionnaire with the other scales that consider a month as the time frame.

*IPSS-10.* The Italian 10-item version of the Perceived Stress Scale (IPSS-10) [38] was used to evaluate athletes' perceived stress. This is a scale based on Lazarus's theory of stress appraisal and measures the extent to which life's aspects are perceived as uncontrollable, unpredictable, and overloading [35]. In particular, the IPSS-10 assesses individual's thoughts and feelings related to stressful events that occurred in the last month. It consists of six negatively stated items (e.g., In the last month, how often have you been/felt unable to control the important things in your life?) and four positively stated items (e.g., In the last month, how often have you been/felt on top of things?) scored on a 5-point Likert scale ranging from 0 (never) to 4 (very often). Scores are calculated reversing positive items scores and then summing up all scores, with a total score ranging from 0 to 40. Higher scores indicate higher levels of perceived stress.

*TQR scale.* The Total Quality Recovery (TQR) scale was used to assess athletes' perceived quality of recovery the morning after YN sessions. This scale was developed to enable an individualized measurement of the perceived recovery process in sport [39]. Specifically, athletes’ attention is directed to psychophysiological cues (mood states and bodily signals, such as sensations of soreness and heaviness). Scores range from 6 to 20. The verbal anchors are: 7 (very very poor recovery), 9 (very poor recovery), 11 (poor recovery), 13 (reasonable recovery), 15 (good recovery), 17 (very good recovery), 19 (very very good recovery). No verbal anchors are used for 6, 8, 10, 12, 14, 16, 18, 10.

*CR-10 Scale.* The CR-10 Scale [45] was used to measure athletes’ daily ratings of perceived exertion (RPE) after training and competition. The use of this scale is considered crucial in reducing ceiling effects. The scale has been used in relation to different physiologic parameters (e.g., maximal oxygen consumption, lactate, and heart rate) [63,64]. Scores range from 0 (no effort) to • (maximal sustainable effort). The verbal anchors are: 0 (nothing at all), 0.5 (extremely weak), 1 (very weak), 2 (weak), 3 (moderate), 5 (strong), 7 (very strong), 10 (extremely strong), and • (absolute maximum; a score of ≥11 is generally assigned to this anchor). No verbal anchors are used for 4, 6, 8, and 9.

*PSAS*. The Pre-sleep Arousal Scale (PSAS; Italian adaptation) [40] was used to assess pre-sleep arousal levels in the last month. This self-report questionnaire consists of 16 items for evaluating somatic (eight items) and cognitive (eight items) arousal experienced at bedtime while attempting to fall asleep. Items reflect manifestations of cognitive (e.g., worry about falling asleep, being mentally alert, can't shut off thoughts) and somatic arousal (e.g., heart racing, muscle tension, rapid breathing) as distinguished modes. Participants are asked to describe how intensely they generally experience each symptom as they attempt to fall asleep in their own bedroom by selecting an appropriate rating of: 1, not at all; 2, slightly; 3, moderately; 4, a lot; 5, extremely. The two subscale scores ranging from 8 to 40 are computed separately by summing the cognitive and somatic arousal items. Overall, cut-off scores of ≥14 and ≥ 20 for the somatic and cognitive subscale respectively indicate higher probability to develop insomnia and insomnia related issues [61].

PSQI. The Italian adaptation of the Pittsburgh Sleep Quality Index (PSQI) [41] was administered to investigate athletes’ sleep quality over the previous month. It is a retrospective self-report questionnaire consisting of 19 items. Seven domains of sleep difficulties (sleep quality, sleep latency, sleep duration, habitual sleep efficiency, sleep disturbances, use of sleeping medications, and daytime dysfunction) are assessed. Considered together, these sleep domains are scored as a single factor of global sleep quality. Usually, a PSQI global score >5 indicates a poor subjective sleep quality. In our study we relied on the global score.

*FS and FAS*. The Feeling Scale (FS) [43] and the Felt Arousal Scale (FAS) [44] were used to easily, and reliably capture athletes' basic affective responses to each YN session, depicting the participant's affective valence (perceived pleasure/displeasure) and arousal (perceived activation), respectively [65]. The feeling scale ranges from −5 (very bad) to +5 (very good), while the felt arousal scale ranges from 1 (low arousal) to 6 (high arousal). Of note, high/low levels of arousal can have both a positive (energy/calmness) or negative (anxiety/boredom) connotation.

*MAIA.* The Italian 32-item version of the Multidimensional Assessment of Interoceptive Awareness questionnaire (MAIA) [66] was used to assess IA. This questionnaire measures “the ability to recognize subtle bodily signals as a controlled state of sustained attention to events that happen within the body here-and-now” (p. 3)^66^. Participants are requested to rate “how often each statement applies to you generally in daily life” on a 6-points Likert scale ranging from 0 (never) to 5 (always). MAIA also consists of eight scales called: (1) Noticing, the simple awareness of one's body sensations (4 items); (2) Not-distracting, the tendency not to ignore or distract oneself from sensations of pain or discomfort (three items); (3) Not-worrying, the tendency not to experience emotional distress or worry with sensations of pain or discomfort (three items); (4) Attention regulation, the ability to sustain and control attention to body sensation (seven items); (5) Emotional awareness, the awareness of the connection between body sensations and emotional states (five items); (6) Self-regulation, the ability to regulate psychological distress by attention to body sensations (four items); (7) Body listening, the tendency to actively listen to the body for insight (three items); and (8) Trusting, the experience of one's body as safe and trustworthy (three items). Based on recent literature on the relationship between perceived stress and IA in athletes [35], in this study we considered Attention Regulation, Emotional awareness, Self-regulation and, in particular, Trusting. Each scale score can vary from 0 to 5 and is calculated by averaging the scores of individual items.

### 5.2 Actigraphy-based sleep assessment

Athletes wore a wrist activity monitor, the ActiGraph wGT3X-bt (Actigraph, Pensacola, USA) to record their sleep parameters. Activity monitors have been deemed a valid alternative to polysomnography to monitor sleep particularly in athletic populations [67]. Placement at the wrist rather than the hip provides better measures of sleep characteristics [68]. For logistical reasons, the actigraph monitoring lasted 5 days before the intervention, after the YN programme and at follow up. ActiGraphs were set for players’ height, weight, date of birth, and nondominant wrist. Recordings were set at 60 s epochs and at a sampling rate of 60 Hz. Adjustable wrist straps were used to minimize discomfort and disruption to sleep. When athletes received the ActiGraph, verbal instructions on how to use it were given, along with printed instructions. They were advised to wear the ActiGraph on their nondominant wrist for 5 consecutive days before commencing the YN programme, at the end of the YN program and three weeks later, returning the ActiGraph to the researcher at the end of each phase. Along with the ActiGraph, athletes received a sleep diary to record bedtime and wake up time. Data derived from the sleep diaries and wrist activity monitors were used to determine the amount and quality of sleep. At the end of each phase, data were downloaded and analyzed using ActiLife software, version 6.13.4. The Cole-Kripke algorithm was used to determine the following sleep parameters: Latency, sleep efficiency (SE), total sleep time (TST), wake after sleep onset (WASO), number of awakenings, average awakening length.

### 5.3 EEG recordings

Electroencephalographic data were recorded using the FlexComp Infinity encoder (Thought Technology Ltd, Montreal, Canada), connected to a laptop computer equipped with Biograph Infinity software (version 6.2, Thought Technology Ltd, Montreal, Canada). The EEG signal was collected through one silver-silver chloride electrode attached to the skull. EEG data were continuously recorded with 2048 Hz sampling frequency. Electrode positioning was monopolar, with the active electrode positioned at Cz. Mastoid reference electrodes were placed on both athlete's earlobes. Electrode skin contact was checked and impedance was kept below 10 kΩ. Data were sampled while athletes were sitting in a quiet environment at a constant room temperature and while they performed a stress test. Each test started with a baseline phase (2 min), that is, a black cross at the center of a blank screen, which athletes were instructed to fixate. Baseline was followed by a stress phase (2 min) with a mental arithmetic test and then a recovery phase (2 min) where athletes had to stare at a cross in the center of a blank screen. The software used contains an assessment program which applies an analogue-to-digital converter to sample the unfiltered analogue EEG and alter it into a digitized signal. This signal is then filtered with a fast Fourier transformation (FFT) in order to produce a digitally filtered signal. The outcome is then amplified by a digital-to-analogue converter to produce the final filtered analogue signal. The same software contains a function that allows for the auto-rejection of artefacts, which was set at ±50 μV. IAPF was identified as the center of gravity frequency within the 8–12 Hz band, and the software continuously calculated iAPF values every second.

### 5.4 Procedure

Participants were recruited using our university networks linked to karate. They were provided with general information on the purposes of the study, while specific information was omitted to avoid biased responses to the questionnaires. Athletes attended a 10-session programme (lasting approximately half an hour for 2 sessions a week), delivered across one-month intervention period (from the end of March to the end of April 2022). Each YN session started with body parts awareness followed by breathing awareness exercises. Then, Sankalpa was introduced asking athletes to pay attention to the feelings and emotions that the statements aroused. This part of the session was followed by visualization exercises where athletes could imagine places evoking feelings of calm. At the end, breathing exercises, body part awareness and mental repetition of one's Sankalpa were repeated. Bell sounds were used to maintain athletes' auditory channel receptive throughout the practice. Each session was pre-recorded by a YN expert, uploaded to SoundCloud (Stockholm, Sweden), and sent via link to athletes' phones after their training so they could listen to the audio track before going to sleep. Before starting the programme, the athletes were provided with FS and FAS. Before and after each YN session athletes were requested to send their responses regarding FS and FAS to the first author of this manuscript, including how they were experiencing arousal levels (i.e., if they were anxious, bored etc.). Participants were also encouraged to practice directly in their bedroom or in a quiet and safe environment to maintain their comfort.

All questionnaires were administered through an online survey platform (i.e., Google Forms) five days prior to the beginning (T0) of the programme, five days after the conclusion (T1), and three weeks later (T2). The follow-up assessment (i.e., T2) was conducted to assess the degree to which the observed effects persisted over time shortly after the implementation of the intervention [60]. On the day of the first assessment (i.e., T0), athletes signed the informed consent and completed a demographic information form before accessing the questionnaires. The same day athletes also accessed and completed MAIA. TQR and CR-10 administration was part of athletes' training routine; specifically, these scales were administered daily by athletes' coach from the 5th of March to the 31st of May, the period corresponding to the qualification for the upcoming world championship. At T0, T1 and T2 athletes also underwent an EEG assessment which took place in a quiet room of athletes’ training facilities. Given the effects of alcohol and caffeinated drinks on brain electrical activity, athletes were instructed not to drink these substances in the last 24 h before the EEG data collection [69].

### 5.5 Statistical analysis

Descriptive statistics (i.e., sum and mean scores) were computed on the questionnaires administered before the intervention, after the intervention and at follow-up. The EEG data were subjected to inferential analysis. The iAPF values were exported with a sampling frequency of 16 Hz, namely, 16 samples per second. Thus, since EEG data collection lasted 6 min, we were able to conduct analysis of variance (ANOVA) on a number of approximately 5.760 samples (∼1920 samples in 2 min). The distribution of the collected iAPF values was Gaussian. Hence, we performed a series of one-way ANOVAs using Bonferroni correction for post hoc pairwise comparisons to statistically compare iAPF values across the three task phases (i.e., baseline, stress and recovery) for each time of data collection (i.e., before intervention, after intervention and at follow up), for each athlete. The sphericity assumption was evaluated using the Mauchly test. Greenhouse–Geisser corrections for degrees of freedom were applied in case of non-sphericity. In the analysis of variance, the effect sizes were calculated using partial eta square (η_p_^2^) [70], while in the case of multiple comparisons, the effect sizes were calculated using Cohen's *d* [70,71]. For each computed ANOVA, the significance level was set at 0.05. Statistical analysis was performed using the Statistical Package for Social Sciences software (SPSS v. 26, IBM, Armonk, United States).

## Data availability statement

Data are not shared publicly to ensure confidentiality. Anonymized data will be made available upon request.

## CRediT authorship contribution statement

**Selenia di Fronso:** Writing - review & editing, Writing - original draft, Supervision, Software, Resources, Project administration, Methodology, Investigation, Formal analysis, Data curation, Conceptualization. **Claudio Robazza:** Writing - review & editing, Supervision, Methodology, Conceptualization. **Dario Pompa:** Writing - review & editing. **Maurizio Bertollo:** Writing - review & editing, Methodology, Conceptualization, Supervision.

## Declaration of competing interest

The authors declare that they have no known competing financial interests or personal relationships that could have appeared to influence the work reported in this paper.
