# Supplementary material for: Dreaming while awake: The beneficial effects of yoga Nidra on mental and physical recovery in two elite karate athletes
Source: Heliyon. 2024 Jan 5;10(1):e24180. doi: 10.1016/j.heliyon.2024.e24180 (PMC10806354; doi:10.1016/j.heliyon.2024.e24180)
Supplement: Multimedia component 1 [file mmc1.docx]

**Supplementary materials**

Table 1S. Ratings of perceived exertion (RPE) and total quality recovery (TQR) values in subject 1 and subject 2 from the 5^Th^ of March to the 31^st^ of May 2022

|  | **Subject 1** | | **Subject 2** | |
| --- | --- | --- | --- | --- |
| **Days** | **RPE** | **TQR** | **RPE** | **TQR** |
| 5-March | 3 | / | 5 | / |
| 6-March | 3 | 13 | 5 | 13 |
| 7-March | 3 | 13 | 5 | 13 |
| 8-March | 3 | 13 | 6 | 12 |
| 9-March | 4 | 13 | 8 | 13 |
| 10-March | 2 | 14 | 3 | 13 |
| 11-March | No training | 15 | No training | 14 |
| 12-March | 4 | 14 | 4 | 14 |
| 13-March | 3 | 14 | 7 | 13 |
| 14-March | 3 | 13 | 6 | 13 |
| 15-March | 3 | 14 | 5 | 13 |
| 16-March | 3 | 15 | 3 | 14 |
| 17-March | 2 | 15 | 2 | 14 |
| 18-March | No training | 13 | No training | 13 |
| 19-March | 3 | 16 | 3 | 15 |
| 20-March | 5 | 14 | 7 | 13 |
| 21-March | 3.5 | 14 | 7 | 14 |
| 22-March | 4 | 13 | 4 | 13 |
| 23-March | 6 | 13 | 6 | 13 |
| 24-March | 7 | 15 | 6 | 14 |
| 25-March | No training | 17 | No training | 16 |
| 26-March | 5 | 18 | 5 | 19 |
| 27-March | 4 | 18 | 7 | 19 |
| 28-March | 6 | 19 | 5 | 19 |
| 29-March | 6 | 19 | 4 | 18 |
| 30-March | 7 | 18 | 4 | 18 |
| 31-March | 4 | 18 | 7 | 18 |
| 1-April | No training | 19 | No training | 20 |
| 2-April | 3.5 | 18 | 3 | 18 |
| 3-April | 6 | 18 | 3.5 | 18 |
| 4-April | 7 | 20 | 6 | 19 |
| 5-April | 6 | 18 | 7 | 18 |
| 6-April | 8 | 18 | 8 | 18 |
| 7-April | 2 | 18 | 3.5 | 18 |
| 8-April | Competition | 19 | Competition | 20 |
| 9-April | No training | 18 | No training | 18 |
| 10-April | 4 | 19 | 4 | 19 |
| 11-April | 5 | 19 | 6 | 20 |
| 12-April | 8 | 19 | 8 | 19 |
| 13-April | 8 | 19 | 9 | 18 |
| 14-April | 7 | 19 | 4 | 19 |
| 15-April | 3.5 | 20 | 3.5 | 20 |
| 16-April | Competition | 19 | Competition | 19 |
| 17-April | Competition | 19 | Competition | 19 |
| 18-April | No training | 19 | No training | 19 |
| 19-April | 3.5 | 19 | 4 | 18 |
| 20-April | 6 | 18 | 7 | 18 |
| 21-April | 5 | 19 | 9 | 19 |
| 22-April | 9 | 20 | 7 | 20 |
| 23-April | 3 | 20 | 3 | 19 |
| 24-April | Competition | 18 | Competition | 18 |
| 25-April | Competition | 20 | Competition | 17 |
| 26-April | 2 | 19 | 2 | 18 |
| 27-April | 7 | 20 | 10 | 18 |
| 28-April | 8 | 13 | 7 | 12 |
| 29-April | 5 | 16 | 5 | 16 |
| 30-April | Competition | 13 | Competition | 12 |
| 1-May | No training | 13 | No training | 13 |
| 2-May | 4 | 14 | 4 | 12 |
| 3-May | 8 | 13 | 7 | 15 |
| 4-May | 6 | 13 | 5 | 11 |
| 5-May | 5 | 13 | 5 | 13 |
| 6-May | 8 | 13 | 7 | 13 |
| 7-May | 2 | 13 | 3.5 | 13 |
| 8-May | No training | 13 | No training | 13 |
| 9-May | 5 | 13 | 5 | 13 |
| 10-May | 5 | 13 | 6 | 16 |
| 11-May | 6 | 12 | 9 | 12 |
| 12-May | 8 | 12 | 8 | 12 |
| 13-May | 3 | 13 | 6 | 13 |
| 14-May | 2 | 13 | 2 | 13 |
| 15-May | Competition | 14 | Competition | 14 |
| 16-May | Competition | 12 | Competition | 15 |
| 17-May | No training | 13 | No training | 12 |
| 18-May | 3.5 | 13 | 4 | 15 |
| 19-May | 4 | 13 | 4 | 12 |
| 20-May | 6 | 13 | 4 | 13 |
| 21-May | 7 | 14 | 8 | 14 |
| 22-May | 3.5 | 14 | 3.5 | 13 |
| 23-May | 3 | 13 | 4 | 13 |
| 24-May | No training | 12 | No training | 12 |
| 25-May | 3 | 12 | 3.5 | 12 |
| 26-May | 2 | 13 | 4 | 13 |
| 27-May | 6 | 13 | 6 | 15 |
| 28-May | 3 | 14 | 4 | 14 |
| 29-May | 4 | 13 | 4 | 14 |
| 30-May | 3 | 13 | 3.5 | 13 |
| 31-May | No training | 13 | No training | 13 |

**Note.** RPE ranges from 0 (no effort) to • (a score of ≥11 is assigned to this anchor; maximal sustainable effort). TQR ranges from 6 to 20. The verbal anchors are: 7 (very very poor recovery), 9 (very poor recovery), 11 (poor recovery), 13 (reasonable recovery), 15 (good recovery), 17 (very good recovery), 19 (very very good recovery). No verbal anchors are used for 6, 8, 10, 12, 14, 16, 18, 10. / = TQR scores are referred to the morning after training; RPE were collected immediately after trainings (which had a mean length of ~90 minutes). Subject 1 = Male elite karate athlete, 36yo; Subject 2 = Female elite karate athlete, 19yo. Red color indicates when yoga nidra was administered
